# The Change of Life in Health and Disease

**Published:** 1857-07

**Authors:** 


ART. X.—*The Change of Life in Health and Disease.* By EDWARD JOHN TILT, M.D. 8vo. pp. 307. Second Edition, London, 1857.

THE comparatively short time which has elapsed since the appearance of the first edition of Dr. Tilt's work on the physiology, and the diseases incident to women between the fortieth and fiftieth years, before a second is called for, is some evidence of the high estimation in which it is held by the profession of Great Britain. The force of this evidence, however, is somewhat weakened by an attempt upon the part of the author to give a somewhat popular air to his production, an attempt, however sure it is always to fail in reference to the professed objects of the author, namely, the enlightenment of the public, is certain to add largely to the sale of the work, to say nothing of its influence upon the practice of such benevolent gentlemen.

But notwithstanding this unprofessional leaning, the book of Dr. Tilt is a valuable addition to our literature upon the subject treated of, and may be appealed to as containing nearly if not quite all that is known concerning not only the physiology, but the pathology, therapeutics, and hygiene of the critical period. Many of the tables are especially valuable, the results drawn from one of which may be new to most of our readers. We refer to the statement in reference to protracted menstruation and fecundity, in which the author has established the fact "that of 10,000 pregnant women, 436 were upwards of forty years of age; and of these 51 exceeded the age of forty-five, and 3 the age of fifty; one being fifty-two, another fifty-three, and the third, fifty-four."

By far the most valuable chapter in the work is that on the "General Principles of Treatment at the Change of Life," which it would be well for many practitioners to study, more especially those who are in the habit of being guided in these matters by the "old wimmen" of their respective neighborhoods.

